# Supplementary figures and images for: Different prevalence and spectrum of malignancy between Chinese patients and American patients with rheumatoid arthritis: a comparative study
Source: PeerJ. 2024 Dec 18;12:e18650. doi: 10.7717/peerj.18650 (PMC11662904; doi:10.7717/peerj.18650)

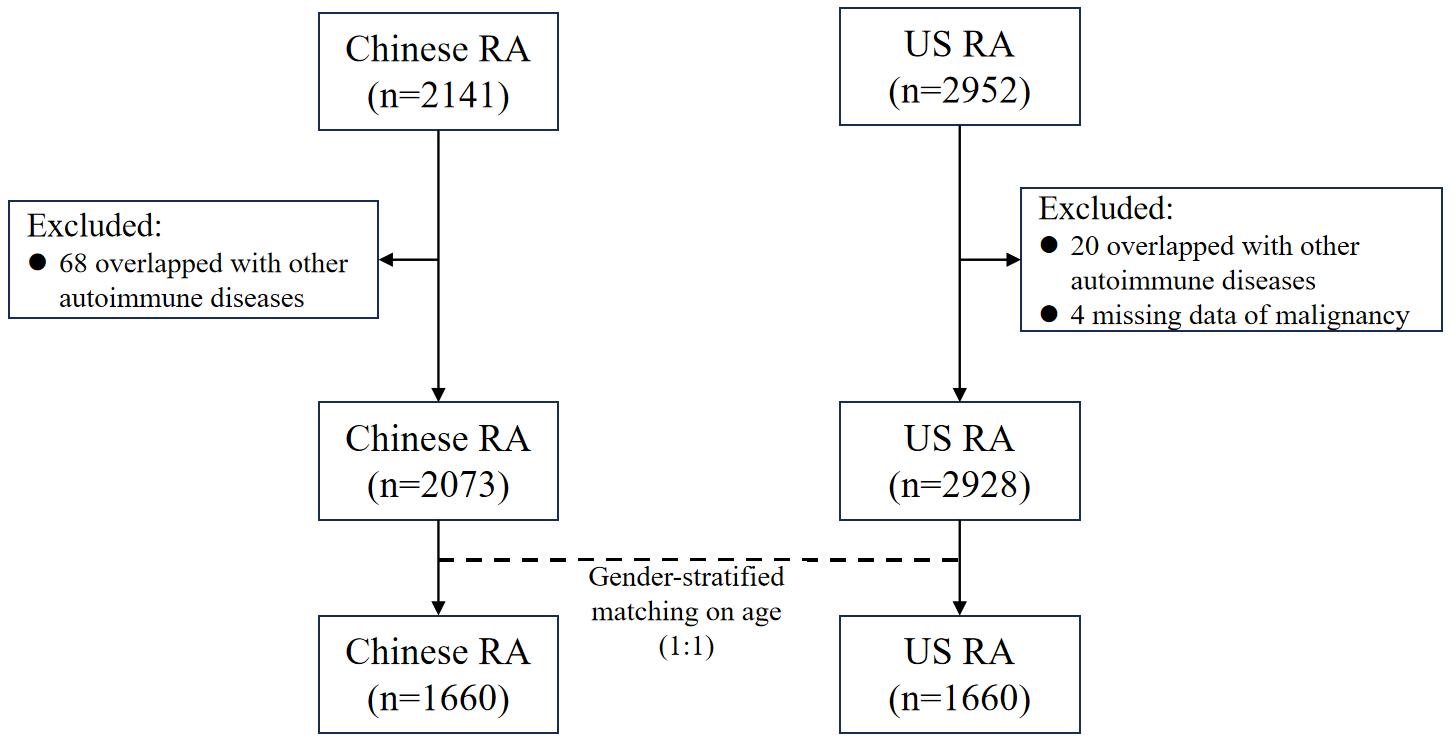

Supplement: Supplemental Information 3 — RA, rheumatoid arthritis. [file peerj-12-18650-s003.jpg]
