# Supplementary material for: Different prevalence and spectrum of malignancy between Chinese patients and American patients with rheumatoid arthritis: a comparative study
Source: PeerJ. 2024 Dec 18;12:e18650. doi: 10.7717/peerj.18650 (PMC11662904; doi:10.7717/peerj.18650)
Supplement: Supplemental Information 4 — RA, rheumatoid arthritis; RF, rheumatoid factor; ACPA, anti-cyclic citrullinated peptide antibody; 28TJC, 28-joint tender joint counts; 28SJC, 28-joint swollen joint counts; PtGA, patient global assessment of disease activity; PrGA, provider global assessment of disease activity; Pain VAS, pain visual analog scale; ESR, erythrocyte sedimentation rate; CRP, C-reactive protein; CDAI, Clinical Disease Activity Index; HAQ-DI, health assessment questionnaire disability index. [file peerj-12-18650-s004.docx]

**Supplemental Table S1 The clinical characteristics of Chinese patients with malignancy after RA onset**

|  | Malignancy before RA onset (n=43) | Malignancy after RA onset (n=75) | *P* |
| --- | --- | --- | --- |
| Female, n (%) | 32 (74.4) | 50 (66.7) | 0.379 |
| Age, years | 59.0 ± 8.8 | 61.0 ± 11.3 | 0.323 |
| RA disease duration, months | 22 (0, 78) | 120 (29, 192) | **＜0.001** |
| Active smoking, n (%) | 8 (18.6) | 23 (30.7) | 0.152 |
| BMI^2^, kg/m^2^ | 22.0 ± 3.2 | 22.0 ± 3.0 | 0.977 |
| Positive RF, n (%) | 33 (76.7) | 69 (92.0) | **0.02** |
| Positive ACPA, n (%) | 36 (83.7) | 68 (90.7) | 0.261 |
| Disease activity indicators |  |  |  |
| 28TJC | 4 (1, 7) | 5 (2, 9) | 0.183 |
| 28SJC | 2 (0, 5) | 3 (1, 6) | 0.180 |
| PtGA | 2 (1, 4) | 4 (1, 6) | **0.032** |
| PrGA | 2 (1, 4) | 4 (1, 5) | **0.035** |
| Pain VAS | 2 (1, 4) | 3 (1, 5) | **0.013** |
| ESR, mm/h | 27 (15, 54) | 52 (22, 83) | **0.020** |
| CRP, mg/L | 4.5 (3.3, 37.9) | 14.9 (3.6, 38.0) | 0.145 |
| CDAI | 12 (5, 18) | 16 (9, 22) | **0.039** |
| HAQ-DI | 0.3 (0, 0.6) | 0.6 (0.3, 1.2) | **0.001** |
| Comorbidity, n (%) |  |  |  |
| Hypertension | 10 (23.3) | 24 (32.0) | 0.313 |
| Diabetes | 1 (2.3) | 16 (21.3) | **0.005** |
| [Dyslipidemia](javascript:;) | 11 (25.6) | 23 (30.7) | 0.557 |
| Cardiovascular diseases | 4 (9.3) | 7 (9.3) | 0.996 |

RA, rheumatoid arthritis; RF, rheumatoid factor; ACPA, anti-cyclic citrullinated peptide antibody; 28TJC, 28-joint tender joint counts; 28SJC, 28-joint swollen joint counts; PtGA, patient global assessment of disease activity; PrGA, provider global assessment of disease activity; Pain VAS, pain visual analog scale; ESR, erythrocyte sedimentation rate; CRP, C-reactive protein; CDAI, Clinical Disease Activity Index; HAQ-DI, health assessment questionnaire disability index.
