# Supplementary material for: Different prevalence and spectrum of malignancy between Chinese patients and American patients with rheumatoid arthritis: a comparative study
Source: PeerJ. 2024 Dec 18;12:e18650. doi: 10.7717/peerj.18650 (PMC11662904; doi:10.7717/peerj.18650)
Supplement: Supplemental Information 5 — Model 1: Unadjusted; Model 2: Adjusted for age, gender (male or female); Model 3: Adjusted for model 2 covariates plus BMI, active smoking (yes or no), CRP, hypertension (yes or no), diabetes (yes or no), dyslipidemia (yes or no), cardiovascular diseases (yes or no), enrollment year interval and previous medication. [file peerj-12-18650-s005.docx]

**Supplemental Table S2 Association of different country with malignancy in the overall cohort in an adjusted model**

| **Characteristics** | Model 1 | |  | Model 2 | |  | Model 3 | |
| --- | --- | --- | --- | --- | --- | --- | --- | --- |
|  | *OR (95% CI)* | *P* |  | *OR (95% CI)* | *P* |  | *OR (95% CI)* | *P* |
| **All RA** |  |  |  |  |  |  |  |  |
| Chinese RA vs. US RA | 0.336 (0.272, 0.416) | **<0.001** |  | 0.445 (0.356, 0.556) | **<0.001** |  | 0.615 (0.438, 0.862) | **0.005** |
| **Male RA** |  |  |  |  |  |  |  |  |
| Chinese RA vs. US RA | 0.535 (0.367, 0.780) | **0.001** |  | 0.795 (0.535, 1.182) | 0.257 |  | 0.962 (0.545, 1.698) | 0.895 |
| **Female RA** |  |  |  |  |  |  |  |  |
| Chinese RA vs. US RA | 0.280 (0.217, 0.363) | **<0.001** |  | 0.340 (0.261, 0.444) | **<0.001** |  | 0.489 (0.320, 0.748) | **<0.001** |

Model 1: Unadjusted; Model 2: Adjusted for age, gender (male or female); Model 3: Adjusted for model 2 covariates plus BMI, active smoking (yes or no), CRP, hypertension (yes or no), diabetes (yes or no), [dyslipidemia](javascript:;) (yes or no), cardiovascular diseases (yes or no), enrollment year interval and previous medication.
